# Electroactive Microbial Consortium of *Bacillus*, *Lysinibacillus*, and *Lactococcus* for Enhanced Wastewater Treatment and Bioelectricity Generation

**DOI:** 10.3390/biology15020124

**Published:** 2026-01-09

**Authors:** Aliya Temirbekova, Zhanar Tekebayeva, Timoth Mkilima, Kamshat Kulzhanova, Zhadyrassyn Nurbekova, Aslan Temirkhanov, Kulyash Meiramkulova, Zhandarbek Bekshin, Akhan Abzhalelov

**Affiliations:** 1 Republican Collection of Microorganisms, Astana 010000, Kazakhstan; 2 Department of General Biology and Genomics, L.N. Gumilyov Eurasian National University, Astana 010000, Kazakhstan; 3 Department of Environmental Engineering and Management, The University of Dodoma, 1 Benjamin Mkapa Road, Iyumbu, Dodoma 41218, Tanzania; 4 Department of Biotechnology and Microbiology, L.N. Gumilyov Eurasian National University, Astana 010000, Kazakhstan; 5 Department of Management and Engineering in the Field of Environmental Protection, L.N. Gumilyov Eurasian National University, Astana 010000, Kazakhstan

**Keywords:** bioelectrochemical systems, chemical oxygen demand removal, synergistic microbial interactions, electrogenic performance optimisation, poultry wastewater biodegradation

## Abstract

Microbial fuel cell (MFC) technology represents a promising bioelectrochemical approach for the simultaneous generation of electricity and treatment of high-strength wastewater. However, the performance of MFCs strongly depends on the metabolic potential and synergistic interactions of the inoculated microbial community. This study evaluated the electrochemical activity and COD removal efficiency of three individual bacterial strains, *Lysinibacillus sphericus* A1, *Bacillus cereus* A2 and *Lactococcus lactis* A4, compared with a developed consortium under long-term operation using poultry slaughterhouse wastewater as a substrate. All inocula were tested in dual-chamber MFCs for 30 days, and performance indicators included power output, voltage, and removal of chemical oxygen demand (COD). The consortium showed the highest power of 170 mW/m^2^ and the optimal voltage–current ratio at a current of 900 mA/m^2^ and 245 mV under decreasing external resistance from 1000 to 50 Ω. The highest COD removal (84.4%) was also recorded, surpassing all pure cultures and demonstrating a significant improvement compared with *B. cereus* A2 and *L. lactis* A4. Meanwhile, the lowest power of 52 mA/m^2^ was recorded during testing of *L. lactis* A4, at 650 mA/m^2^ and 120 mV. Compared with single cultures, the consortium produced approximately 15% higher power density than *L. sphericus* A1, about 29% higher than *B. cereus* A2, and more than threefold higher than *L. lactis* A4. This study highlights the potential of a consortium as an efficient biocatalyst for MFC-mediated wastewater treatment and suggests that selecting complementary strains with diverse metabolic functions can substantially improve system performance.

## Simple Summary

Cleaning polluted water while also generating renewable electricity is a major scientific goal. One promising approach uses helpful bacteria that can break down waste in water and release electrons, which can be captured as electrical energy. In this study, we tested three different types of naturally occurring bacteria, both individually and combined as a group, to see how well they could clean very dirty water from poultry processing and how much electricity they could produce at the same time. Each type of bacteria was placed in a device that produces electricity directly from biological activity, and their performance was monitored for one month. The study found that the group of bacteria working together produced more electricity and removed more pollution from the water than any of the bacteria tested alone. The mixed group cleaned about 84% of the waste in the water, which was higher than the cleaning ability of each single type. It also produced the strongest electrical output. These results show that using several bacteria with different strengths can greatly improve both water cleaning and electricity generation. This approach may help to develop more sustainable technologies for treating industrial wastewater while recovering useful energy.

## 1. Introduction

Over the past two decades, the ecotoxicological impact of industrial wastewater has become a major concern, underscoring the need for effective removal of pollutants to mitigate environmental harm. Traditional treatment methods have proven to be energy-intensive and economically inefficient, which has prompted increasing interest in more sustainable, non-conventional technologies, including bioelectrochemical systems [1,2,3,4,5,6]. The removal of organic compounds from wastewater by anodic oxidation has been proposed by many researchers as an energy-efficient treatment technology [7,8]. Microbial fuel cell (MFC) technology is a promising approach to generate electricity using microorganisms that convert chemical energy into electrical energy [9]. In a typical MFC setup, electrons released by anodic biofilm bacteria after oxidation of an electron donor (typically an organic substrate) are first transferred to the anode under anoxic conditions [10]. This indicates that the anode is used by the electrogenic biofilm bacteria as the electron acceptor for anaerobic respiration. Protons generated from the oxidation process migrate to the cathode, usually through a proton exchange membrane (PEM) that prevents diffusion of oxygen into the anodic chamber. Electrons reach the cathode via an external circuit with an applied resistance load [11]. The electrogenic capacity of MFCs generally depends on strains, substrates, electrode properties, and operating conditions [12]. For the successful operation of a microbial fuel cell, it is important to characterise the biofilm on the anode [13]. The behaviour of MFCs during initial biofilm growth and characterisation of anodic biofilm were studied using two-chamber MFCs with activated sludge as inoculum [14].

Pure cultures are useful for elucidating the electron transfer mechanism at the microbiological level and further reducing the number of specific microbial strains in mixed cultures. The perfect symbiotic relationship of mixed cultures could establish a perfect direct interspecies electron transfer, which leads to an increase in MFC efficiency. Various pure electrogenic bacteria, such as *Geobacter* sp., *Enterobacter*, and *Shewanella* sp., have been studied as biocatalysts. Microbial fuel cells have also been developed with bacteria like *Escherichia coli*, *Serratia marcescens*, *Bacillus* spp., *Pseudomonas aeruginosa*, etc. [15]. *Bacillus amyloliquefaciens* NSB4 can be effectively utilised as an inexpensive and highly efficient exoelectrogen in single-chamber MFC technology without the use of external mediators [16]. *B. thuringiensis* STV1324a and *B. aquimaris* STV1324b generated electricity with maximum power densities of 351.24 mW/m^2^ and 442.8 mW/m^2^, respectively [17]. However, some recent studies reported the production of a higher current density from wastewater, using mixed culture. Microbial consortium transfers more electrons to the anode and degrades the organic compound more efficiently than pure cultures [18,19].

Numerous studies have convincingly demonstrated that the electrogenic performance and adaptability of microbial fuel cells inoculated with pure cultures are significantly inferior to those of systems based on mixed microbial consortia [20,21,22]. A key factor underlying their effectiveness is the pronounced synergistic effect that emerges from the interactions between different microorganisms [23]. The effectiveness of mixed microbial communities has been consistently confirmed through experimental evidence [24,25,26,27,28,29,30].

A consortium of strains with different metabolic potentials, suitable for microbial fuel cells (MFCs), represents a new and important approach that could significantly advance the development of this field. The combination of metabolic capabilities can lead to more efficient substrate degradation and increased energy yield. In addition, different types of microorganisms can complement each other in electrochemical interactions with electrodes in MFCs. For example, *B. cereus* can provide high electron transfer [31], while *L. sphaericus* can provide effective interaction with the electrode surface or electroactivation [32]. This can improve the overall performance of MFC by optimising the electron transfer and electrogeneration processes. In addition, various species of *Lactococcus*, which metabolise a wide range of organic and inorganic substances [33], may be candidates for effective MFC, suggesting synergy with the above strains in MFC performance [34]. These microorganisms are highly tolerant to a wide range of environmental conditions, making them suitable for use in MFC under various climatic and operating conditions. In addition, their ability to be activated by different types of substrates and cultivation conditions makes them promising candidates for use in MFC with diverse nutrient sources. *L. sphaericus* has the additional ability to process toxic substances, such as insecticides, that may be present in wastewater or other types of MFC substrates. This may make the consortium of *B. cereus* and *L. sphaericus* particularly useful for cleaning contaminated aquatic environments while simultaneously generating electricity.

In this study, three strains were selected from the previously studied electrogenic strains—*Lysinibacillus sphericus* A1, *Bacillus cereus* A2, and *Lactococcus lactis* A4—isolated from wastewater from a poultry farm. The selection of the key parameters of the experimental design was based on previous studies [35] involving the listed strains, in which their voltage generation was evaluated in dual-chamber MFCs using simulated poultry farm wastewater under a fixed external resistance of 100 Ω. The study aims to compare the electroactivity of these strains in pure form and in mixed culture, as well as to study their potential for treating wastewater with high organic pollution.

## 2. Materials and Methods

### 2.1. Experimental Materials and Instruments

#### 2.1.1. Selection of Electroactive Strains and Preparation of Inoculums

Three electroactive bacterial strains were used in this study: *Lysinibacillus sphaericus* A1, *Bacillus cereus* A2, and *Lactococcus lactis* A4. These strains were originally isolated from poultry farm wastewater in previous work, and their electrogenic activity in wastewater-fed microbial fuel cells has been reported previously [35]. All cultures were preserved in long-term storage at the Republican Collection of Microorganisms in Astana. Prior to experimentation, each strain was revived on nutrient meat broth (HiMedia Laboratories Pvt. Ltd., Mumbai, India) and incubated at 37 °C for 24 h. For inoculum preparation, the strains were cultivated in 10% Luria–Bertani broth (HiMedia Laboratories Pvt. Ltd., Mumbai, India) on a rotary shaker at 150 rpm and 37 °C overnight. The resulting cultures were adjusted to an optical density, OD_600_, of 0.8 ± 0.05 to standardise initial cell concentrations across all treatments. A volume of 1 mL of each culture was introduced into the anode chambers of the MFCs, either individually or as an equal-volume mixture to form the microbial consortium. All inoculum preparations were performed in triplicate to ensure experimental reproducibility. In addition, a sterile, uninoculated anolyte prepared under identical conditions was operated in parallel as a control to account for background electrochemical signals and non-biological changes in substrate composition. This control did not include any microbial inoculum and was used solely for comparative purposes under the same amended and sterilised conditions.

#### 2.1.2. Anolyte Substrate

Poultry slaughterhouse wastewater was collected at the drainage point prior to any on-site treatment at the Izhevski facility in Astana and used as a base matrix for anolyte preparation. Samples were transported on ice and stored at 4 °C for less than 24 h before processing. To establish a controlled and reproducible substrate for comparative laboratory experiments, the wastewater was supplemented with glucose (3 g/L; HiMedia Laboratories Pvt. Ltd., Mumbai, India), tryptone (10 g/L; TM Media, India), and yeast extract (5 g/L; Condalab, Madrid, Spain) before use. The pH of the amended wastewater was adjusted to 7.0 ± 0.1. The basic physicochemical properties of the wastewater base matrix, including chemical oxygen demand (COD), pH, electrical conductivity, total suspended solids, and volatile suspended solids, were measured before sterilisation, as summarised in Table 1. Following characterisation, the amended wastewater was sterilised by autoclaving at 121 °C for 20 min to eliminate indigenous microorganisms and ensure that electrochemical activity in the MFCs originated from the defined inocula. The resulting anolyte therefore represents a nutrient-enriched and sterilised model substrate, rather than untreated slaughterhouse wastewater.

#### 2.1.3. Catholyte Composition

The cathode chamber was filled with a 50 mM phosphate buffer that was adjusted to pH 7.0 to provide a stable chemical environment throughout the experimental period. Potassium ferricyanide was added at a concentration of 50 mM and served as the terminal electron acceptor in the cathodic compartment. The use of ferricyanide was intended to minimise cathodic limitations and to provide a consistent and well-defined redox for laboratory scale performance comparison. Prior to sealing the chamber, the catholyte was gently aerated for 10 min to ensure homogeneous distribution of dissolved oxygen and to establish reproducible initial redox conditions across all replicates. To avoid depletion of the electron acceptor or changes in oxidative capacity during operation, the catholyte was replaced every 72 h. This replacement protocol helped maintain stable cathodic conditions and ensured that the observed variations in voltage and power output were primarily associated with anodic bioelectrochemical activity, rather than changes in catholyte composition. These conditions provided a controlled cathodic environment that was suitable for comparative evaluation of the electrogenic performance of the different microbial treatments.

#### 2.1.4. Proton Exchange Membrane (Nafion) Pretreatment

Nafion 117 proton exchange membranes were prepared through a standard activation procedure designed to optimise their proton conductivity and long-term stability within the microbial fuel cell. The membranes were first boiled for 1 h in a 3% H_2_O_2_ solution, which removed organic residues and surface contaminants that could interfere with ion transport. Following this step, they were rinsed thoroughly with ultrapure water to eliminate the remaining traces of peroxide. The membranes were then boiled for another 1 h in 0.5 M H_2_SO_4_ to recondition the sulfonic acid groups and restore full proton-exchange capacity. After acid treatment, the membranes were again rinsed with ultrapure water until no residual acid remained. They were stored in ultrapure water prior to assembly to maintain hydration and flexibility, both of which are essential for proper membrane function. This pretreatment protocol ensured that the membrane contributed consistently to ion transport without introducing variability due to surface fouling or reduced conductivity.

#### 2.1.5. Sterilisation of Electrodes and MFC Components

Graphite plates with dimensions of 5 × 2 cm were used as both the anode and cathode electrodes in all microbial fuel cell units. Prior to assembly, each plate was thoroughly washed with ethanol to remove oils, dust, and other surface contaminants that could interfere with biofilm attachment or electrical performance. The electrodes were subsequently placed in an ultrasonic bath containing deionised water and sonicated for 10 min to dislodge the remaining particles. Following cleaning, the electrodes were sterilised by autoclaving at 121 °C for 20 min to minimise the introduction of external microorganisms into the experimental system. All additional components, including acrylic chambers, tubing, seals, and connectors, were disinfected with 70% ethanol and dried completely with a laminar flow hood prior to use. These preparation steps were implemented to reduce contamination risk and to ensure that the electrochemical activity observed during operation was primarily associated with the intentionally inoculated bacterial cultures under controlled laboratory conditions. Consistent sterilisation across all reactors contributed to experimental accuracy and reproducibility among replicates.

### 2.2. MFC Experimental Setup

**Figure 1:**
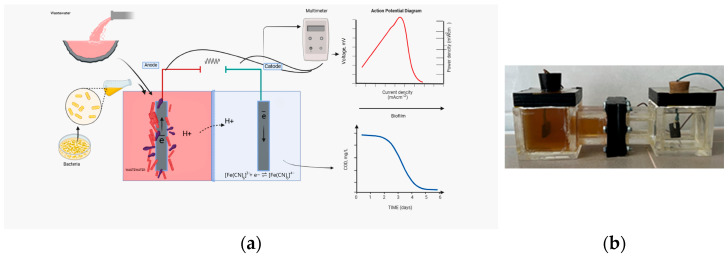
Experimental setup. (**a**) Scheme of process from inoculum preparation to MFC testing for electricity generation and wastewater treatment efficiency and (**b**) dual-chamber MFC.

A dual-chamber microbial fuel cell (MFC) was employed for all experimental runs, and the general configuration is illustrated in Figure 1. The reactor was constructed from acrylic glass with internal dimensions of 10 × 10 × 5 cm, providing a working volume of approximately 450 mL for both the anode and cathode compartments. The chambers were separated by a pretreated Nafion 117 proton exchange membrane, which allowed proton transport while preventing direct mixing of the anolyte and catholyte. Each chamber included a headspace of approximately 50 mL to accommodate gas accumulation and pressure fluctuations during batch operation. To minimise airborne contamination and reduce evaporation, the chambers were sealed with Parafilm and covered with aluminium foil. Graphite plates were installed in both compartments as electrodes, and connected externally using copper wires and crocodile clips [36]. A fixed external resistance of 100 Ω was incorporated into the circuit to enable continuous voltage monitoring. Due to the small reactor volume and batch mode operation, this configuration was intended for a controlled laboratory-scale comparison of electrochemical performance, rather than a simulation of full-scale wastewater treatment systems. To maintain the constant activity of the bacterial strains, the temperature was kept between 30 and 35 °C.

The anode chamber was made anaerobic by flushing with N_2_ gas for 5 min before sealing, which created an oxygen-limited environment and promoted electron transfer to the anode rather than to dissolved oxygen. Voltage output was continuously recorded using a UK-831LN digital multimeter (China) to allow for fine-scale monitoring of electrical performance throughout the experimental period. Each treatment, including individual strains, the mixed consortium, and the sterile control, was tested using three independent MFC units. Replication ensured that the results could be evaluated statistically and that differences between treatments reflected biological and electrochemical factors, rather than random variation. This standardised setup provided a reproducible platform for comparing the effects of microbial composition on MFC performance.

### 2.3. Analytical Methods

#### 2.3.1. Electrochemical Testing

Cell voltage (V) was measured every 24 h for a total period of 30 d, allowing a detailed analysis of the temporal progression of electrochemical performance. Current (I) was calculated using Ohm’s law, based on the measured voltage and the imposed external resistance of 100 Ω. Power density (mW/m^2^) was then calculated using the standard formula that incorporates voltage, current, and the anode surface area, which was 0.001 m^2^. These calculations allowed for comparison of power output between treatments, independent of electrode size or volume. To obtain a more detailed understanding of the electrochemical behaviour, polarisation and power density curves were constructed using five external resistances (50, 100, 200, 500, and 1000 ohms), resulting in five steady-state data points. Each resistance was held for 20 min to allow the voltage to stabilise before measurements were recorded. This procedure provided information on the internal resistance, maximum power output (Equation (1)), and electron transfer characteristics of the microbial communities. The combination of long-term monitoring and polarisation analysis allowed a thorough evaluation of MFC performance across all treatments.

(1) $$P(mW/m^{2})=\frac{V\times I}{A}\times 1000$$

Whereby V (mV) is the output voltage, I (mA/m^2^) is the current density, and A (m^2^) is the anode surface area.

#### 2.3.2. Analysis of COD Removal Efficiency

Chemical oxygen demand (COD) was measured using Hach Lange LCK 514 digestion vials with a detection range of 100 to 2000 mg/L. Prior to digestion, samples were filtered through a 0.45 µm membrane to remove suspended solids that could interfere with optical measurements. When necessary, samples were diluted to ensure that COD concentrations were within the analytical range of the vials. Digestion was carried out at 150 °C for 2 h, after which the samples were cooled and analysed at 600 nm, using a Hach DR3900 spectrophotometer (Hach, Berlin, Germany). All measurements were performed in triplicate, and are reported as mean values with standard deviations. COD removal was calculated based on the difference between initial and final COD concentrations. It should be noted that, in this study, COD removal reflects changes in the total oxidisable organic content of the amended and sterilised anolyte. This does not distinguish between microbial degradation of native wastewater organics, consumption of supplemented substrates, biomass assimilation, or abiotic processes. Therefore, COD removal is used solely as a comparative indicator of substrate utilisation among the different microbial treatments, rather than a definitive measure of wastewater treatment performance (Equation (2)).

(2) $$COD\;removal\;\left(\%\right)=\frac{C_{i}{-C}_{f}}{C_{i}}\times 100$$

Whereby C*_i_* and C_f_ are the initial and final COD (g/L), respectively [37].

Analysis of all the samples has been performed in triplicate, and standard deviation has been incorporated.

#### 2.3.3. SEM Analysis of Biofilm Formation

Biofilm formation on the anode surfaces was examined using a Zeiss Crossbeam 540 scanning electron microscope (Carl Zeiss Microscopy GmbH, Oberkochen, Germany). After MFC operation, the anodes were removed and fixed in a solution containing 2.5% paraformaldehyde and 1.5% glutaraldehyde, prepared in 0.1 M cacodylate buffer at pH 7.5. Fixation was carried out for 12 h at 4 °C to preserve the microbial structure and attachment. After fixation, the electrodes were rinsed twice with buffer and dehydrated through a graded ethanol series from 30% to 100%. Critical point drying was used to prevent the collapse of delicate biofilm structures. The dried electrodes were sputter-coated with a 10 nm layer of Au/Pd to enhance conductivity during imaging. SEM observations were performed at a 5 to 10 kV accelerating voltage and an 8 to 15 mm working distance. Magnifications from 500× to 10,000× were used to examine both large-scale biofilm coverage and fine structural features. This approach allowed for a detailed comparison of biofilm morphology across the different microbial treatments.

#### 2.3.4. Statistical Analysis

All data were analysed using OriginPro 2022. One-way ANOVA was used to compare differences in MFC performance across the individual strains and the consortium. A significance threshold of *p* < 0.05 was used to determine statistically meaningful differences. The use of triplicate independent MFCs for each treatment ensured that the statistical analysis reflected biological and operational reproducibility. This analytical approach provided rigorous evaluation of variations in power density, voltage output, and COD removal efficiency. The combined statistical framework supported a valid interpretation of the experimental findings and strengthened the reliability of the conclusions drawn from the study.

## 3. Results

### 3.1. Physicochemical Characteristics of Poultry Wastewater Samples Before Testing in MFC

The collected poultry farm wastewater was characterised to provide baseline information on its physicochemical properties. Visually, the wastewater exhibited a muddy red colour and a noticeable hydrogen sulphide odour, which are commonly observed in poultry effluents and indicate the presence of substantial organic matter. These qualitative observations provide context regarding the composition of the wastewater prior to any laboratory treatment and establish the conditions under which the experiments were conducted. Quantitative measurements of the wastewater showed a pH value of 7.6, indicating a slightly alkaline condition that is typical for livestock-associated effluents. The chemical oxygen demand (COD) was measured at 2150 mg L^−1^, reflecting a high level of oxidizable organic matter in the collected sample. The average temperature of the wastewater was approximately 15 °C at the time of collection, which may influence microbial activity, although the laboratory experiments were performed under controlled conditions at 37 °C. It should be emphasised that these characteristics represent the raw, collected wastewater prior to any modifications. For the microbial fuel cell experiments, the wastewater was sterilised and supplemented with nutrients to create a controlled laboratory environment. Therefore, the measured physicochemical parameters provide background information on the original sample and serve as a reference for interpreting experimental outcomes, without implying direct treatment performance or biodegradability in the unmodified wastewater.

### 3.2. Testing Electroactive Bacterial Cultures and Their Consortium Double-Chambered MFC for Electricity Generation

**Figure 2:**
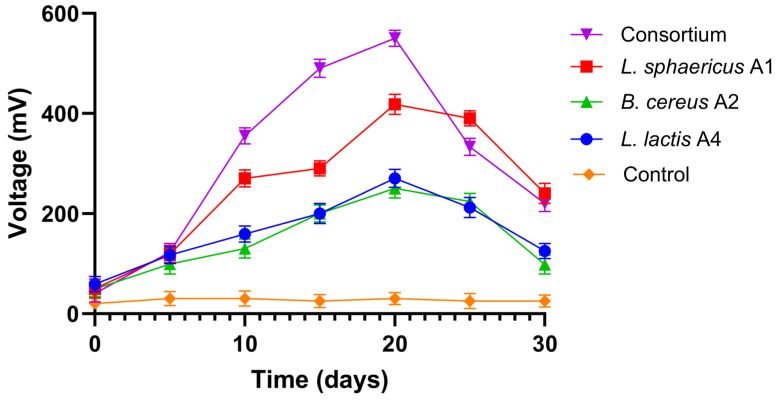
Voltage generation of the MFC for the strains *L. sphaericus* A1, *B. cereus* A2, *L. lactis* A4, their consortia, and abiotic control (sterile wastewater).

In the first stage of the bioelectrochemical studies, the study analysed the voltage generation dynamics for pure cultures—*L. sphaericus* A1, *B. cereus* A2 and *L. lactis* A4—and their consortium. As shown in Figure 2, this study compared individual electrogenic bacterial strains, *L. sphaericus* A1, *B. cereus* A2, and *L. lactis* A4, and their consortium in terms of voltage generation in a two-chamber microbial fuel cell. Among the strains, *L. sphaericus* A1 stood out, generating a maximum voltage of 418 ± 15 mV. After 20 days, the voltage of *L. sphaericus* A1 decreased more slowly than that of all bacterial cultures and a voltage of 240 ± 15 mV was still achieved in the end. The second electroactive isolate, *B. cereus* A2, showed a maximum power density of 270 ± 13 mV over 30 days. *L. lactis* A4 showed the lowest voltage of 270 ± 1 mV in MFC, generating 8% less voltage than *L. sphaericus* A1, but in the consortium, it provided an additional amplifying effect. The voltages of the strain increased in the initial 20 days and then decreased gradually until the end of the operation. The consortium showed a sharp increase in voltage generation, indicating a synergistic interaction between the strains. Analysis of the data showed that on days 16–20 of the two-chamber MFC test, the mixed culture had the greatest effect on voltage generation compared to the individual strains. After 20 days of testing with *L. sphaericus* A1 and the consortium, the voltage remained above 300 mV, whereas in the test with strains *B. cereus* A2 and *L. lactis* A4, it was below 250 mV. As a result, for all tested strains, three operational phases can be distinguished: the initial adaptation phase, the active phase, and the decline phase. The observed differences between the experimental groups after day 20 likely reflect a different balance between metabolic intensity, substrate utilisation efficiency, and biofilm stability. The consortium prioritised high initial productivity due to combined metabolic activity, while *L. sphaericus* A1 demonstrated greater long-term electrochemical stability. Generally, the pure culture bacteria took a longer time to achieve significant voltage generation compared to the consortium in MFC. The results of the 30-day test are shown in Figure 2.

**Figure 3:**
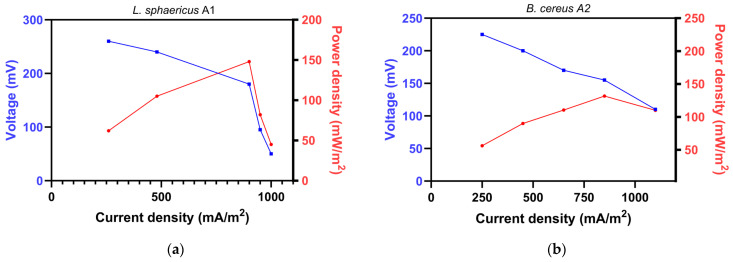

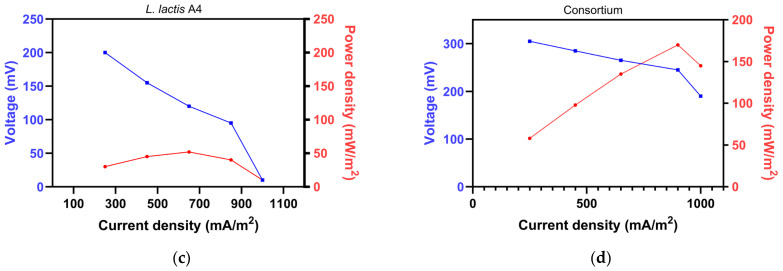
Polarisation curves of the MFC for the strains (**a**) *L. sphaericus* A1, (**b**) *B. cereus* A2, (**c**) *L. lactis* A4, and (**d**) their consortium, obtained under a series of external resistances of 1000, 500, 200, 100, and 50 Ω.

In this study, for MFCs inoculated with single cultures and mixed cultures, a polarisation curve was generated to evaluate the relationship between resistance and current during fuel cell operation (Figure 3). As the external resistance decreased (from 1000 to 50 Ω), the current density increased for all systems, accompanied by a gradual decrease in cell voltage. The MFC inoculated with the designed microbial consortium demonstrated the highest power output, achieving a maximum power density of 170 mW/m^2^ at a current density of approximately 900 mA/m^2^ (Figure 3d). The polarisation behaviour showed a gradual voltage decrease with increasing current density within the ohmic region, followed by a more pronounced voltage decline at higher current densities, indicating the onset of mass transport limitations. The lowest performance was observed for *L. lactis* A4 (Figure 3c), with a maximum power density of only 52 mW/m^2^, accompanied by a rapid voltage decline at higher current densities. *L. sphaericus* A1 (Figure 3a) demonstrated the highest electrochemical activity among individual strains (148 mW/m^2^), indicating effective adaptation to the anode surface of the MFC. However, at lower external resistances, a more pronounced voltage drop was observed compared with the consortium, suggesting limited tolerance to higher current densities. *B. cereus* A2 (Figure 3b) exhibited moderate electrochemical activity (131.8 mW/m^2^), which was substantially lower than that of *L. sphaericus* A1. The power output declined more rapidly with increasing current density, indicating a lower tolerance to electrochemical loading. Overall, the consortium outperformed *L. sphericus* A1 by approximately 15% and *B. cereus* A2 by 29% and exhibited more than a threefold increase in power density compared with *L. lactis* A4.

### 3.3. COD Removal Efficiency

**Figure 4:**
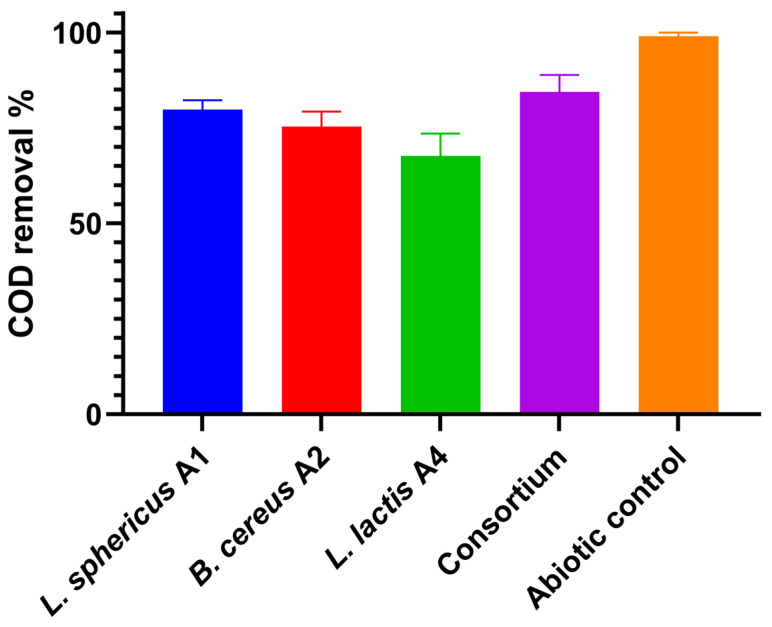
COD removal efficiency using different bacterial cultures in MFC.

The effect of different bacterial inoculants on the chemical oxygen demand (COD) of poultry wastewater was evaluated in the anode chamber of the microbial fuel cell (Figure 4). The initial COD concentration of the wastewater was 2150 mg/L. Single cultures of *Lysinibacillus sphaericus* A1, *Bacillus cereus* A2, and *Lactococcus lactis* mg/L A4, as well as their designed consortium, were tested to determine their efficiency in degrading added organic substrates over a 30-day operational period. Figure 3 presents the COD removal performance of the individual strains and the consortium, while Figure 4 shows the corresponding biofilm formation on the anode surfaces, illustrating the relationship between microbial attachment and substrate degradation. After 30 days, the consortium demonstrated the highest COD removal efficiency, significantly outperforming the single cultures. *L. sphaericus* A1 and *B. cereus* A2 exhibited moderate removal, while *L. lactis* A4 showed the lowest COD reduction among the individual strains. The observed trends suggest that synergistic interactions among the microbial species in the consortium enhanced the overall metabolic activity, leading to more effective substrate utilisation and electron transfer to the anode. Statistical analysis using one-way ANOVA revealed that the differences in COD removal between the treatments were highly significant (*p* < 0.05). A post hoc Tukey’s test confirmed that the consortium achieved a significantly greater COD reduction than any of the single strains, whereas differences among *L. sphaericus* A1, *B. cereus* A2, and *L. lactis* A4 were also significant but less pronounced. These results underscore the advantage of using mixed microbial communities in MFC applications for high-strength wastewater treatment.

The highest COD removal efficiency was 84.4 ± 4.5% in MFC inoculated with a consortium, where the concentration of added organic substrates decreased from 2150 ± 60 mg/L to 436 ± 21 mg/L. *L. sphaericus* A1 is close to the consortium in terms of efficiency, 79.7± 2.5%, with a decrease in COD to 335 ± 19 mg/L, respectively. This is explained by the consortium’s property of having a more diverse metabolism than pure cultures. The minimum COD removal of 67.7 ± 5.8% was observed with a pure culture of *L. lactis* A4, which is most likely due to the limited degradation of complex substrates. Wastewater from poultry farms likely requires mixed microbial cultures.

### 3.4. SEM Analysis of the Anodic Biofilm and Morphology of Electroactive Strains

**Figure 5:**
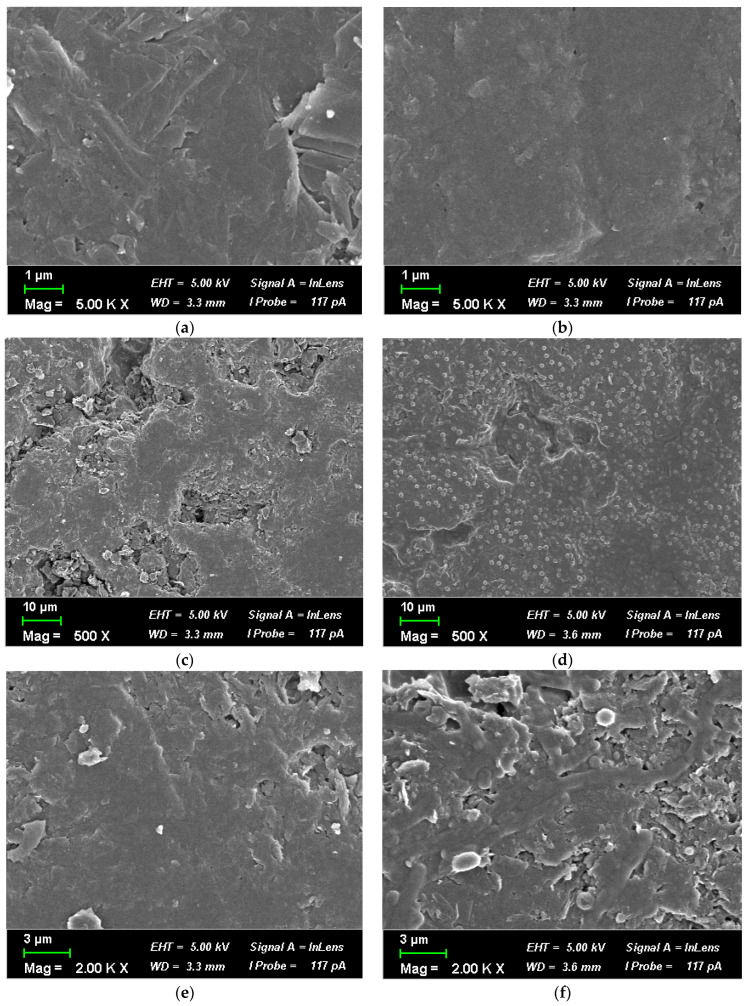
SEM analysis of formation of biofilms of potential electroactive bacterial cultures on the anode in a two-chamber MFC: (**a**) abiotic control before 30 days, (**b**) abiotic control after 30 days, (**c**) *L. lactis* before 30 days, (**d**) *L. lactis* after 30 days, (**e**) consortium before 30 days, and (**f**) consortium after 30 days.

Figure 5 shows the SEM images of the biofilm within the MFC reactor before and after biofilm formation on the surface of the electrode inside the MFC reactor. The graphite electrodes of the anode chamber were scanned on the 1st and 30th days of the incubation period. The images obtained showed that microorganisms grew on the carbon surface (anode) in the form of an attached biofilm. Before the experiment, the absence of microbial biofilm was recorded on the graphite electrode removed from the MFC anode chamber, which was confirmed visually and by SEM analysis. On day 30, biofilm formation was observed, and the anode surface was completely covered with bacterial cells, as shown in Figure 5. Figure 5 shows a pure culture of *L. lactis* A4 (Figure 5a) and the consortium (Figure 5b) of all three strains. SEM analysis helped confirm the formation of a biofilm in samples with *L. lactis* A4 and the consortium, which shows a direct link between electricity generation in a two-chamber MFC. It was found that the biofilm with the consortium has the thickest matrix layer. The biofilm formation process correlated with the voltage and current dynamics in the two-chamber MFC, which was a consequence of the influence of increasing electrical parameters—voltage and current. *L. lactis A4* attached to graphite had a uniform oval morphology. This study has provided a clue about bacterial behaviour both in a pure culture and in a mixed culture from inside the microbial fuel cell.

**Figure 6:**
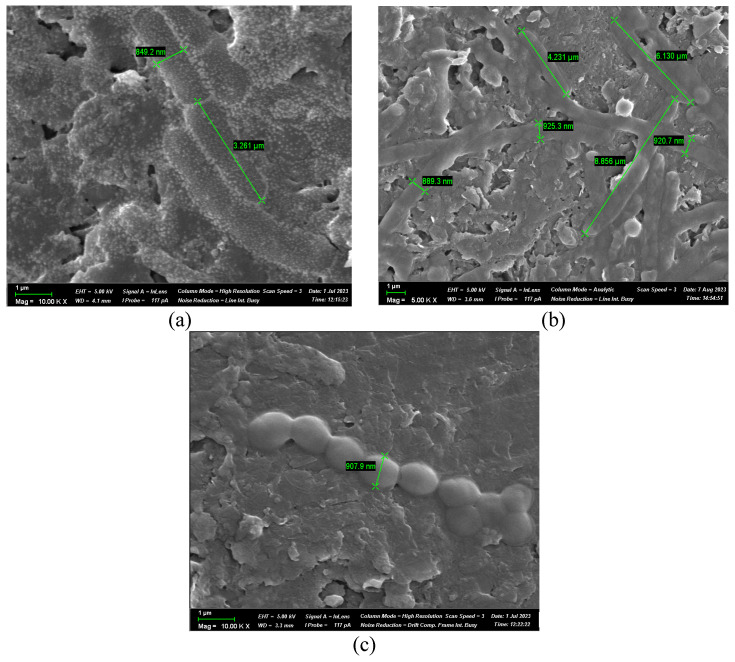
SEM analysis of electroactive bacterial strains ((**a**)—*L. sphaericus* A1, (**b**)—*B. cereus* A2, and (**c**)—*L. lactis* A4).

SEM (Figure 6) images of individual bacterial strains on the anode surface illustrate cell morphology and size characteristics: *L. sphaericus* A1 cells with a width of approximately 0.8–0.9 µm and a length of 4–6 µm; *B. cereus* A2 cells measuring about 0.9 µm in width and 3.3 µm in length; and *L. lactis* A4 coccoid cells with an average diameter of 0.9 µm.

**Figure 7:**
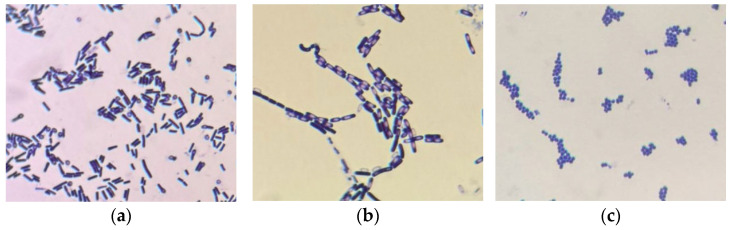
Microscopy of electroactive strains of consortium ((**a**)—*L. sphaericus* A1, (**b**)—*B. cereus* A2, and (**c**)—*L. lactis* A4).

Microscopy was performed after activation of the electroactive strains that were previously preserved in a cryogenic medium. The strains exhibited morphological characteristics (Figure 7) that were typical for their respective species, which had earlier been identified based on 16 S rRNA analysis.

### 3.5. Comparative Analysis

From Table 2, it can be observed that mixed microbial cultures generally exhibited relatively high-power outputs. In particular, one mixed culture achieved a power density of 465.3 ± 5.8 mW/m^2^, which was approximately 6.8 times higher than that of the pure culture tested in parallel. However, there are also reported cases in which mixed cultures exhibited lower electrochemical activity (120 mW/m^2^) compared with *B. subtilis* generating 270 mW/m^2^. These data indicate that pure cultures, in certain cases, produce power outputs that are comparable to or even exceed those of mixed cultures. According to the comparative data in Table 1, the consortium developed in this study is inferior to the listed pure cultures but prevails over some *Shewanella* species, such as *B. amyloliquefaciens* NSB4. However, it should be noted that in this study, anodic electrodes with minimised characteristics (volume and surface area) were employed. Among the strains listed above (Table 2), *L. sphaericus* A1 and *B. cereus* A2 demonstrated intermediate electrochemical activity and high COD removal efficiencies. Although COD is a convenient integral parameter for assessing organic matter degradation, it does not reflect the full complexity of biodegradation processes. The lack of BOD, TOC, and VFA indicators should be considered a limitation of this study and should be taken into account when interpreting the results. In future studies, it would be advisable to include an expanded set of biodegradation indicators and an improved system of control experiments for a comprehensive assessment of organic matter transformation efficiency.

## 4. Discussion

The efficiency of electricity generation and COD removal is considered to be an indicator for assessing the stable operation of MFC. In this study, three strains were selected—*L. sphericus* A1, *B. cereus* A2, and *L. lactis* A4—which were previously isolated from poultry farm wastewater. The maximum capacity of the MFC inoculated with the consortium suggests a lower apparent internal resistance and improved electrochemical behaviour of the consortium compared with single-strain systems. The consortium displayed a stable polarisation profile with a monotonic voltage decrease and a well-defined power density maximum (170 mW/m^2^ at 900 mA/m^2^), indicating efficient electron transfer and enhanced electrochemical stability across the tested resistance range. The of *L. lactis* A4 in wastewater suggests possible natural compatibility with *L. sphericus* A1 and *B. cereus* A2. This combination ensures potential metabolic complementarity, structural stability of the biofilm, and an increase in electron flow, due to interspecies interaction. However, the effectiveness of each strain in isolation and its contribution to the mixed culture have not been fully studied. Some studies show that pure *Bacillus* cultures may have a limited ability to generate electricity in MFCs, due to low electron transfer rates or insufficient electrogenesis activity [38,39]. This may limit their practical application in energy systems. Some *Bacillus* strains may exhibit insufficient resistance to changes in MFC operating conditions, such as changes in pH, temperature, or nutrient concentration [40]. This can lead to reduced productivity or uneven MFC performance in the long term. Eliminating these limitations and gaps may require additional research to better understand the biological and electrochemical mechanisms of strains in MFCs and to develop methods for improving their electrogenic potential and resistance to environmental changes. In this study, the strains did not inhibit but maintained stable surface-associated microbial growth in the consortium, whereas in studies on *B. cereus* in combination with *P. aeruginosa*, inhibition of biofilm formation was found, which was confirmed by its visualisation. It is known that *B. cereus* can effectively use sucrose as an electron donor for electricity generation and can be used for simultaneous wastewater treatment and electricity production, but it depends on external resistance [41]. Moreover, the microbial diversity of the developed consortium contributed to enhanced system stability during long-term operation by sustaining voltage generation through an extended active phase, approximately lasting from day 5 to day 25, compared with the shorter active phases observed for the single strains (Figure 2).

Studies on poultry slaughterhouse wastewater containing bird droppings, feed, feathers, hatchery waste, urine, faeces, sawdust, etc., were found to be suitable for creating an artificial biofilm on the MFC anode. For example, using chicken manure wastewaters as a valuable substrate, an energy collection performance of 278 mW/m^2^ and 82%BOD removal efficiency was reported for a continuous horizontal flow MFC [42,45].

The use of mixed culture in the MFC anode reactor, especially with complex real industrial organic waste, has shown significant potential for generating energy from decomposing organic waste [46]. Therefore, wastewater can be used directly as inoculum for MFCs. Poultry farm wastewater contains organic substrates that are easily decomposed. This is a good source for generating electricity in MFCs. When diluted wastewater from a distillery was inoculated into the anode chamber, the peak power and current density reached 168 mW/m^2^ and 580 mA/m^2^. Co-cultured microorganisms can expand the range of carbon sources in MFCs. Co-cultivation conditions were systematically optimised to balance glucose catabolism and electron transfer. A maximum power density of 123.4 mW/m^2^ was ultimately achieved by this MFC. Thus far, the combination of the two species has not resulted in a significant increase in efficiency as a type of wastewater or activated sludge. Nevertheless, certain co-cultivation MFCs provided a strategy for studying the interaction between different microorganisms in different consortia. The interaction between *G. sulfurreducens* and *E. coli* was studied in their co-cultured MFC. Metabolomic analysis showed that the consumption of both succinate and oxygen by E. coli would contribute to an increase in the current production of *G. sulfurreducens*. In terms of treatment efficiency, the acclimatised MFC culture resulted in approximately 1.5 times higher COD removal than the non-acclimatised MFC culture [47]. Moreover, the microbial fuel cell provided a maximum power density of 1.45 W/m^3^ in the acclimatisation phase and 1.32 W/m^3^ in the treatment phase. The results showed that microbial fuel cells operating on real, highly concentrated dairy wastewater can achieve high efficiency [48].

One of the key aspects is the development of methods for managing and stabilising the consortium in the MFC. This includes optimising environmental conditions, controlling competition between different types of microorganisms, and preventing one species from dominating others. To make MFC technology economically viable, extensive research is needed on the creation of microbial communities from different habitats that can form biofilms and accept electrons from electrodes.

## 5. Conclusions

This study addressed the challenge of improving electricity generation and pollutant removal in microbial fuel cells treating high-strength poultry slaughterhouse wastewater. By comparing three individual bacterial strains with a designed consortium, we demonstrated that mixed microbial communities offer clear performance advantages. The consortium achieved the highest power density of 170 mW/m^2^, the most favourable voltage–current behaviour at 900 mA/m^2^ and 245 mV, and the greatest chemical oxygen demand removal of 84.4%, surpassing all pure cultures. In contrast, the weakest performance was recorded for *L. lactis* A4, which only produced 52 mW/m^2^ at 675 mA/m^2^ and 120 mV. These findings indicate that the microbial consortium exhibited enhanced electrochemical performance compared with the individual strains. The key takeaway from this work is that the combination of multiple strains was associated with improved MFC performance under the tested conditions, supporting the broader goal of recovering energy while treating complex organic waste streams. Overall, the developed consortium represents a promising and sustainable biocatalyst strategy for advancing microbial fuel cell applications. It is crucial, however, to regard the present study as a laboratory proof-of-concept that demonstrates feasibility, rather than providing a realistic evaluation of large-scale performance. The work was conducted under controlled conditions, using a sterilised, amended wastewater matrix in small-scale batch reactors, and its scope was limited by the sole use of COD for assessing treatment efficiency and a 30-day operational period. Therefore, while the results indicate significant functional potential, they highlight the need for future investigations to validate scalability and practical applicability. Subsequent research should transition to non-sterile, real wastewater in larger-scale, continuous-flow systems over extended periods. Furthermore, efforts should focus on elucidating the metabolic mechanisms behind the observed synergies and incorporating a broader suite of analytical parameters (e.g., BOD, TOC, VFAs) to fully understand biodegradation pathways and long-term system stability.

## Figures and Tables

**Table 1:** Physico-chemical characterisation of the wastewater sample.

Physico-Chemical Parameters	Observation
Colour	Muddy red
Odour	Hydrogen sulphide smell
pH	7.6
COD (mg/L)	2150
Temperature (°C Average)	15

**Table 2:** Comparative characterisation of power density and COD removal efficiency of single and mixed microbial cultures in MFCs.

Bacterial Culture	MFC Type	Highest Power Density, mW/m^2^	COD Removal Efficiency	References
*B. amyloliquefaciens* NSB4	Single-chamber MFC	41.281 mW/m^2^	90.46%	[16]
Mixed culture	Single-chambered MFC	465.3 ± 5.8 mW/m^2^	Not available	[38]
Pure culture of Shewanella.	Single-chambered MFC	68.7 ± 3.7 mW/m^2^		
Mixed culture	Dual-chamber MFC	120 mW/m^2^	Not available	[39]
*B. subtilis*	Dual-chamber MFC	270 mW/m^2^	Not available	
*B. cereus*	Dual-chamber MFC	400 mW/m^2^	Not available	[40]
*B. subtilis* BSC-2	Dual-chamber MFC	405 mW/m^2^	Not available	[41]
*B. cereus*	Dual-chamber MFC	185.90 mW/m^2^	82%	[42]
Mixed culture	Dual-chamber MFC	223 ± 11 mW/m^2^	Not available	[43]
*Shewanella baltica* 20	Dual-chamber MFC	12 mW/m^2^	57%	[44]
Consortium of *L. sphericus* A1, *B. cereus* A2, and *L. lactis* A1	Dual-chamber MFC	170 mW/m^2^	84.4 ± 4.5%	this study
*L. sphericus* A1	Dual-chamber MFC	148 mW/m^2^	79.7 ± 2.5%	this study
*B. cereus* A2	Dual-chamber MFC	131 mW/m^2^	75.38 ± 3.9%	this study
*L. lactis* A1	Dual-chamber MFC	52 mW/m^2^	67.7 ± 5.8%	this study

## Data Availability

All the data presented within this manuscript will be available online.
